# A mathematical model for optimizing the indications of liver transplantation in patients with hepatocellular carcinoma

**DOI:** 10.1186/1742-4682-10-60

**Published:** 2013-10-20

**Authors:** Eleazar Chaib, Marcos Amaku, Francisco AB Coutinho, Luis F Lopez, Marcelo N Burattini, Luiz AC D’Albuquerque, Eduardo Massad

**Affiliations:** 1 Department of Gastroenterology, Liver and Pancreas Transplantation Surgery Unit, LIM 37, School of Medicine, University of Sao Paulo, Av.Dr. Arnaldo 455, Sao Paulo CEP 01246-903, Brazil; 2 Department of Medical Informatics, LIM 01, School of Medicine, University of Sao Paulo, Av.Dr. Arnaldo 455, Sao Paulo CEP 01246-903, Brazil

**Keywords:** Liver transplantation, Hepatocellular carcinoma, Expanded criteria, Modelling

## Abstract

**Background:**

The criteria for organ sharing has developed a system that prioritizes liver transplantation (LT) for patients with hepatocellular carcinoma (HCC) who have the highest risk of wait-list mortality. In some countries this model allows patients only within the Milan Criteria (MC, defined by the presence of a single nodule up to 5 cm, up to three nodules none larger than 3 cm, with no evidence of extrahepatic spread or macrovascular invasion) to be evaluated for liver transplantation. This police implies that some patients with HCC slightly more advanced than those allowed by the current strict selection criteria will be excluded, even though LT for these patients might be associated with acceptable long-term outcomes.

**Methods:**

We propose a mathematical approach to study the consequences of relaxing the MC for patients with HCC that do not comply with the current rules for inclusion in the transplantation candidate list. We consider overall 5-years survival rates compatible with the ones reported in the literature. We calculate the best strategy that would minimize the total mortality of the affected population, that is, the total number of people in both groups of HCC patients that die after 5 years of the implementation of the strategy, either by post-transplantation death or by death due to the basic HCC. We illustrate the above analysis with a simulation of a theoretical population of 1,500 HCC patients with tumor size exponentially. The parameter λ obtained from the literature was equal to 0.3. As the total number of patients in these real samples was 327 patients, this implied in an average size of 3.3 cm and a 95% confidence interval of [2.9; 3.7]. The total number of available livers to be grafted was assumed to be 500.

**Results:**

With 1500 patients in the waiting list and 500 grafts available we simulated the total number of deaths in both transplanted and non-transplanted HCC patients after 5 years as a function of the tumor size of transplanted patients. The total number of deaths drops down monotonically with tumor size, reaching a minimum at size equals to 7 cm, increasing from thereafter. With tumor size equals to 10 cm the total mortality is equal to the 5 cm threshold of the Milan criteria.

**Conclusion:**

We concluded that it is possible to include patients with tumor size up to 10 cm without increasing the total mortality of this population.

## Introduction

Liver transplantation (LT) or hepatic transplantation is the replacement of a diseased liver with a healthy liver from another person (allograft) [1]. The most commonly used technique is orthotopic transplantation, in which the native liver is removed and replaced by the donor organ in the same anatomic location as the original liver. In a substantial proportion of patients with liver failure, orthotopic transplantation is the only treatment option [2].

Liver failure occurs when large parts of the liver become damaged beyond repair and the liver is no longer able to function. It may be caused by infections, toxic substances, inherited diseases or malnutrition [3]. The chronic aggression of liver tissue by one of the causes of liver failure can end up in primary hepatocellular carcinoma, a deadly condition to which liver transplantation is the only option, with variable success rate of a close to normal life after the surgery [4].

Within the past 5 years, the proportion of patients with HCC in waiting lists for LT has increase dramatically: this proportion has reached more than 26% across Europe and 34% in the United States [5].

The Milan Criteria, MC, is defined by the presence of a single nodule up to 5 cm, up to three nodules none larger than 3 cm, with no evidence of extrahepatic spread or macrovascular invasion. In the countries that adopt the MClaw allows patients only within MC to be evaluated and considered for LT. This police implies that some patients with HCC slightly more advanced than those allowed by the current strict selection criteria will be excluded, even though LT for these patients might be associated with acceptable long-term outcomes [6-8].

We propose a mathematical approach to study the consequences of relaxing the MC for patients with HCC that do not comply with the current rules for inclusion in the transplantation candidate list. We consider overall 5-years survival rates compatible with the ones reported in the literature. We simulate our model in order to reproduce what is known about the survival of the two groups of patients (those who comply with the strict MC and those who do not) and calculate the best strategy that would minimize the total mortality of the affected population, that is, the total number of people in both group that dies after 5 years of the implementation of the strategy, either by post-transplantation death or by death due to the basic HCC.

## Methods

### The Model

We assumed, as a model, that HCC patients present themselves along a short time interval Δ*T* with tumors of variable sizes. We call this interval "at presentation". During this time interval we assumed that *N* HCC patients are included in the transplantation waiting list, and that *F* livers are available to these patients.

The model is based on four assumptions, namely,

1. the mortality rate of non-transplanted,*α*_*nt*_ and transplanted,α_t_ HCC patients are described by the following ad hoc expressions:

(1) $$\alpha_{\mathrm{nt}}\left(s\right)=\alpha_{0}\left(\alpha_{1}-e^{-\delta_{1}s}\right)$$

And

(2) $$\alpha_{t}\left(s\right)=\alpha_{0}+\delta_{2}s$$

where δ_i_(*i*=1,2) are the parameters, such that *δ*_1_>*δ*_2_and *s* is the size of the tumor. In equation (1), when α_1_=2 the above mortality rates coincide for *s*=0. Since this is necessary, we assume *α*_1_=2 for the rest of the paper. Note that *s* is the size of the tumor at the moment patients get into the transplantation program. So, equations (1) and (2) take into account the fact that tumors grow with time and so does the mortality rates. This is included in a a rather cavalier manner in equations (1) and (2) since the functional relationship of tumors growths related mortality with time are not known.

**Figure 1:**
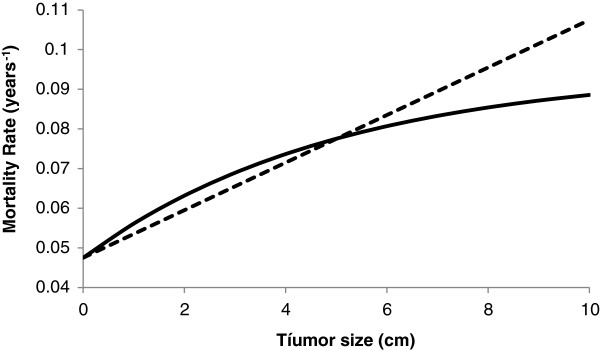
**Mortality rates for transplanted (dotted line) and non-transplanted (solid line) HCC patients.** Results of the theoretical population analyzed, according to equations (1) and (2) with *α*_0_ = 0.048, *δ*_1_ = 0.02 and *δ*_2_ = 0.006.

Equations (1) and (2) are illustrated in Figure 1, in which it is shown the mortality rates for both the transplanted and non-transplanted HCC patients as a function of the tumor size *s* at presentation.

The probability of surviving after *T* years for non-transplanted and transplanted patients, π_*nt*_(*s*) and π_*t*_(*s*), respectively, as a function of their tumor size, *s*, at the time individuals are included in the transplantation program, is given by

(3) $$\pi_{\mathrm{nt}}\left(s\right)=\exp\left(-\alpha_{\mathrm{nt}}T\right)$$

and

(4) $$\pi_{t}\left(s\right)=\exp\left(-\alpha_{t}T\right)$$

Equations (3) and (4) result in survival probabilities after *T* years that are in agreement with data available in the literature. They were used to calculate the form and parameters of equations (1) and (2).

2. the mortality of both transplanted and non-transplanted HCC patients is a monotonically increasing function of tumor size at presentation (tumor size is, therefore, taken as an indication of gravity).

3. the number of available livers to be grafted, *F*, is limited and always less than the total number of HCC, *N*, who have transplantation indication; and finally,

4. the tumor size, *s*, at the time individuals are included in the transplantation program, is distributed in the HCC population according to an exponential distribution, such that the probability that a given HCC patient has tumor size *s* is described by the *probability density function (p.d.f.)*:

(5) $$f\left(s,\lambda \right)=\lambda e^{-\mathrm{\lambda s}}$$

where λ is the *rate parameter* of the distribution. This implies that in a HCC population, many individuals have tumor of small size and a very low number of who present tumors of larger size. Again, this distribution of tumor size is that at the moment the patients get into the transplantation program. The *cumulative distribution function (C.D.F.)* is given by

(6) $$F\left(s,\lambda \right)=\int_{0}^{s}\lambda e^{\mathrm{\lambda t}}\mathrm{dt}=1-e^{\mathrm{\lambda s}}$$

Equation (6) means the probability that a given HCC patient has tumor size equal or less than *s*.

The exponential distribution has mean (*expected value*) equal to:

(7) $$E\left[s\right]=\frac{1}{\lambda }$$

and variance

(8) $$\mathrm{Var}\left[s\right]=\frac{1}{\lambda^{2}}$$

**Figure 2:**
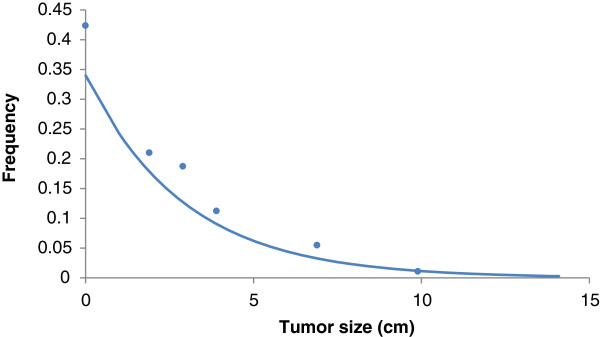
**Frequency distribution of tumor size.** Dots represent actual values from references [9-11] and the line is the exponential fitting to the real data (R^2^ = 0.92). Parameter λ = 0.3 which implies in an average tumor size of 3.3 cm.

In Figure 2 we show the actual distribution of tumor size, fitted to an exponential distribution. The parameter λ in this case is equal to 0.3. As the total number of patients in these samples was 327 patients, this implies in an average size of 3.3 cm and a 95% confidence interval of [2.9; 3.7] [9-11].

With the above assumptions we define *p(s)ds* as the proportion of individuals with tumor size between *s* and *s+ds*; *x(s)ds* the proportion of transplanted patients with tumor size between *s* and *s+ds*; and *y(s)ds* the proportion of non-transplanted patients with tumor size between *s* and *s+ds*. These proportions are related such that:

(9) $$x\left(s\right)=\frac{F}{N}p\left(s\right)$$

and

(10) $$\;y\left(s\right)=\left(1-\frac{F}{N}\right)p\left(s\right)$$

Equations (9) and (10) can be interpreted as follows: a proportion *p(s)* of the HCC patients has tumor size *s*, of which a fraction $\;\frac{F}{N}$is transplanted and its complement $\left(1-\frac{F}{N}\right)$is not transplanted, such that *x*(*s*) + *y*(*s*) = *p*(*s*). Note that, this was a particular transplantation policy. For example, we could replace equation (9) and (10) by$x\left(s\right)=g\left(s\right)\frac{F}{N}p\left(s\right)$ and$\;y\left(s\right)=\left(1-g\left(s\right)\frac{F}{N}\right)p\left(s\right)$, where *g(s)* is some bias towards any eventual tumor size preference. In this work, *g(s)=*1, meaning that all HCC patients have the same chance of being transplanted (no bias). According to the Milan criteria,

$$\;g\left(s\right)=\left\{\begin{array}{l} 1\;\mathrm{if}\;s\leq s_{M}=5\;\mathrm{cm}\\ 0\;\mathrm{if}\;s>s_{M}=5\;\mathrm{cm} \end{array}\right).$$

We then calculated:

1. If we choose to transplant every patient with any tumor size equal or less than a critical tumor size, *S*_*F*_, then to guarantee that all patients with such tumor size less than *S*_*F*_ are transplanted (that is, all grafts are used), *S*_*F*_ has to be defined as:

(11) $$N\int_{0}^{s_{F}}p\left(s\right)\mathrm{ds}=F$$

or

(12) $$s_{F}=-\frac{\log\left(1-\frac{F}{N}\right)}{\lambda }$$

In other words, this means to choose a policy such that *x(s*≤*S*_*F*_*)=p(s)* and *y(s≤S*_*F*_*)=*0.

Equations (11) and (12) can be interpreted as follows: $\int_{0}^{s_{F}}p\left(s\right)\mathrm{ds}$is the fraction of the population that has tumors of size equal or less than *S*_*F*_. Multiplied by the total population *N* gives the total number of individuals that are transplanted, that is, received all the liver grafts *F*. In other words, all available livers are used. The size limit that guarantees that this happens, *S*_*F*_, is therefore calculated as a function of *F* as in equation (12).

2. Hence, if not all patients with tumor size *s* are transplanted, for example, if we choose to transplant $x\left(s\right)=\frac{F}{N}p\left(s\right)$ and not transplant $\;y\left(s\right)=\left(1-\frac{F}{N}\right)p\left(s\right)$, then we can choose to transplant all the patients with tumor size up to *S*_0_>S_F_.

3. Using the Milan criteria (see above), the proportion of non-transplanted patients with tumor size *s* below *S*_*M*_ with respect to the total number of HCC patients at presentation is:

(13) $$p_{\mathrm{nt}}\left(s<s_{M}\right)=\left\{\begin{array}{c} \frac{N\left(1-e^{-\lambda s_{M}}\right)-F}{N}\;\mathrm{if}\;F<N\left(1-e^{-\lambda s_{M}}\right)\;\\ 0\;\mathrm{otherwise}\; \end{array}\right)$$

Equation (13) means that multiplying the proportion of patients with tumor size equal or less than *S*_*M*_, 1-*e*^-λS^_M_ by the total population of HCC, *N*, gives the number of patients with tumors of size up to *S*_*M*_. This number minus the number of available livers, divided by the total population size gives the proportion of non-transplanted patients.

4. The proportion of transplanted patients with respect to the total number of HCC patients at presentation, with tumor size *s* below *S*_*M*_:

(14) $$p_{t}\left(s<s_{M}\right)=\left\{\begin{array}{c} \frac{F}{N}\;\mathrm{if}\;F<N\left(1-e^{-\lambda s_{M}}\right)\;\\ \frac{F}{N}\left(1-e^{-\lambda s_{M}}\right)\;\mathrm{otherwise}\; \end{array}\right)$$

Note that in the exceptional and unique case when *S*_F_=S_M_ all the grafts are used (see Models' Limitations for a more thorough discussion).

Equation (14) reflects the fact that a fraction $\;\frac{F}{N}$of those individuals with tumor size equal or less than *S*_*M*_ is transplanted when the number of available livers *F* is less than the number of individuals with tumor size greater than *S*_*M*_ at presentation.

5. If the Milan criteria is obeyed, then the proportion of non-transplanted patients with tumor size greater than *S*_*M*_:

(15) $$p_{\mathrm{nt}}\left(s>s_{M}\right)=e^{-\lambda s_{M}}$$

which is the minimum (if *F* is not enough to transplant up to *S*_*M*_)proportion of individuals with tumor size greater than *S*_*M*_. According to the Milan criteria none of those patients are transplanted, independently of *F*.

6. If the Milan criteria is not obeyed, then there is a proportion of transplanted patients with tumor size greater than *S*_*M*_ that could be transplanted. This proportion is limited by the number of available livers, and it is:

(16) $$p_{t}\left(s>s_{M}\right)=\left\{\begin{array}{c} \frac{Fe^{-\lambda s_{M}}}{N}\;\mathrm{if}\;F>N\left(1-e^{-\lambda s_{M}}\right)\;\\ 0\;\mathrm{otherwise}\; \end{array}\right)$$

In this situation, the proportion of non-transplanted is given by:

(17) $$p_{t}\left(s>s_{M}\right)=\left\{\begin{array}{c} e^{-\lambda s_{M}}\;\mathrm{if}\;F<N\left(1-e^{-\lambda s_{M}}\right)\;\\ 1-\frac{F}{N}\;\mathrm{otherwise}\; \end{array}\right)$$

Note that adding the proportion of non-transplanted individuals with tumor sizes greater and less than *S*_*M*_ gives$1-\frac{F}{N}$. By the same token, adding the proportion of transplanted individuals with tumor sizes greater and less than *S*_*M*_ gives $\frac{F}{N}$.

7. Now, we abandon the Milan criteria and transplant a proportion $x\left(s\right)=\frac{F}{N}p\left(s\right)$of individuals with tumor size up to *S*_0_ (variable), and compare the impact on the total mortality of HCC patients with the mortality resulting from adopting the Milan criteria.

First we calculate the survival of transplanted patients (*TS*) with tumor size up to *S*_*0*_ at a moment in time *T* after the patients presentation. The proportion of the individuals with tumor size up to *S*_0_ at presentation is:

(18) $$\int_{0}^{s_{0}}\lambda e^{-\mathrm{\lambda s}}\mathrm{ds}$$

The proportion of patients at presentation who were transplanted and survived up to *T* after the transplantation is:

(19) $$\int_{0}^{s_{0}}x\left(s\right)e^{-\alpha_{t}\left(s\right)T}\mathrm{ds}=\frac{F}{N}\int_{0}^{s_{0}}\lambda e^{-\mathrm{\lambda s}}e^{-\alpha_{t}\left(s\right)T}\mathrm{ds}$$

Hence the total number of transplanted patients (*TS*) with tumor size up to *S*_0_ at presentation and who survived up to time *T* is given by Equation (19) multiplied by *N*:

(20) $$\mathrm{TS}=N\frac{F}{N}\int_{0}^{s_{0}}\lambda e^{-\mathrm{\lambda s}}e^{-\alpha_{t}\left(s\right)T}\mathrm{ds}=F\int_{0}^{s_{0}}\lambda e^{-\mathrm{\lambda s}}e^{-\alpha_{t}\left(s\right)T}\mathrm{ds}$$

1. 8. The number of patients with tumors up to tumor size *S*_0_ at presentation who were not transplanted is

(21) $$N\int_{0}^{s_{0}}y\left(s\right)\mathrm{ds}=N\int_{0}^{s_{0}}\left(1-\frac{F}{N}\right)\lambda e^{-\mathrm{\lambda s}}\mathrm{ds}$$

and those who survived after time *T* are:

(22) $$N\int_{0}^{s_{0}}\left(1-\frac{F}{N}\right)\lambda e^{-\mathrm{\lambda s}}e^{-\alpha_{\mathrm{nt}}\left(s\right)T}\mathrm{ds}$$

Now, the number of patients with tumors greater than size*s*_0_at presentation that were not transplanted is:

(23) $$N\int_{s_{0}}^{\infty }p\left(s\right)\mathrm{ds}=N\int_{s_{0}}^{\infty }\lambda e^{-\mathrm{\lambda s}}\mathrm{ds}$$

and, among those, the survivors after time *T* are:

(24) $$N\int_{s_{0}}^{\infty }\lambda e^{-\mathrm{\lambda s}}e^{-\alpha_{\mathrm{nt}}\left(s\right)T}\mathrm{ds}$$

Hence, the total number of survivors after time *T* who were not transplanted is:

(25) $$\mathrm{NTS}=N\int_{0}^{s_{0}}\left(1-\frac{F}{N}\right)\lambda e^{-\mathrm{\lambda s}}e^{-\alpha_{\mathrm{nt}}\left(s\right)T}\mathrm{ds}+N\int_{s_{0}}^{\infty }\lambda e^{-\mathrm{\lambda s}}e^{-\alpha_{\mathrm{nt}}\left(s\right)T}\mathrm{ds}$$

9. Therefore, the Total Survival is obtained by adding equations (20) and (25):

(26) $$\mathrm{Survivors}=F\int_{0}^{s_{0}}\lambda e^{-\mathrm{\lambda s}}e^{-\alpha_{t}\left(s\right)T}\mathrm{ds}+N\int_{0}^{s_{0}}\left(1-\frac{F}{N}\right)\lambda e^{-\mathrm{\lambda s}}e^{-\alpha_{\mathrm{nt}}\left(s\right)T}\mathrm{ds}+N\int_{s_{0}}^{\infty }\lambda e^{-\mathrm{\lambda s}}e^{-\alpha_{\mathrm{nt}}\left(s\right)T}\mathrm{ds}$$

10. Finally, the Total Mortality is given by

(27) $$M\left(s_{0}\right)=N-\left[F\int_{0}^{s_{0}}\lambda e^{-\mathrm{\lambda s}}e^{-\alpha_{t}\left(s\right)T}\mathrm{ds}+N\int_{0}^{s_{0}}\left(1-\frac{F}{N}\right)\lambda e^{-\mathrm{\lambda s}}e^{-\alpha_{\mathrm{nt}}\left(s\right)T}\mathrm{ds}+N\int_{s_{0}}^{\infty }\lambda e^{-\mathrm{\lambda s}}e^{-\alpha_{\mathrm{nt}}\left(s\right)T}\mathrm{ds}\right]$$

11. Now, to calculate the optimal transplantation strategy, we determine the tumor size that can be transplanted and find either *s* such that

min[*M*(*s*)] or *s* such that *M*(*s*)=*M*(*S*_*M*_).

## Results

**Figure 3:**
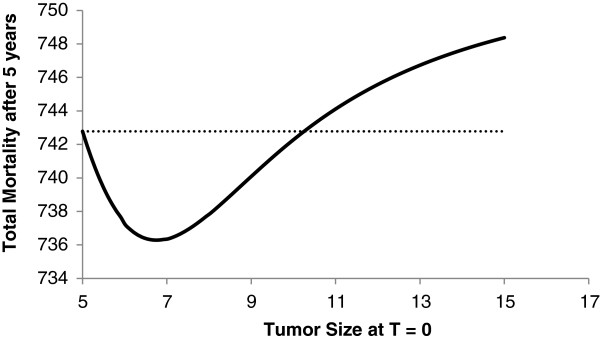
**Total mortality after 5 years comprising both transplanted and non-transplanted HCC patients in a 1,500 theoretical population.** We show only what happens when individuals with tumor size greater that the strict Milan criteria (5 cm).

We illustrate the above analysis for a simulation of a theoretical population of 1,500 HCC patients with tumor size parameter distribution of λ equal to 0.3. As the total number of patients in the real samples from which data was retrieved was 327 patients, this implied in an average size of 3.3 cm and a 95% confidence interval of [2.9; 3.7] [9-11]. The total number of available livers to be grafted was assumed to be 500. With this, we simulated the total number of deaths in both transplanted and non-transplanted HCC patients after 5 years as a function of the tumor size of transplanted patients. The result is shown in Figure 3.

Figure 2 shows the total mortality in the HCC patients cohort, including those transplanted and those non-transplanted as well. The dotted line is a reference line: the point where the mortality curve crosses it is the maximum tumor size that could be transplanted without worsening the mortality in the list. Note that it is possible to include patients with tumor size up to 10 cm without increasing the total mortality of this cohort.

## Discussion

Some limitations of the model should be highlighted. Firstly, the most important, is the fact that we considered a cohort of HCC patient isolated from the others causes of liver failures and, therefore, from the waiting list. We circumvent this by assuming that the 500 available grafts were the equivalent of the number of livers typically allocated to this kind of patients. Secondly, we arbitrarily assumed an exponential distribution for the tumor size, although this is likely to be true. Thirdly, we assumed an *ad hoc* function for the death rates of transplanted and non-transplanted patients. However, assuming any convex function for transplanted mortality rate as a function of tumor size, and concave function for non-transplanted mortality rate would not qualitatively modify our results. Finally, on important consequence of the model, although not directly observable from the equations, is that by transplanting patients with tumor size greater than *S*_*F*_, and, therefore, not transplanting a proportion of patients with tumor size less than *S*_*F*_ may result in a certain proportion of *F* livers that will not be used. This is a consequence of equation (20) when *T*=0, that is, $F\int_{0}^{s_{0}}\lambda e^{-\mathrm{\lambda s}}\mathrm{ds}=F\left(1-e^{-\lambda s_{0}}\right)$. Note, however, that this would happen with any model that would not transplant all the patients below a certain tumor size when there are enough livers available. Equation (14) illustrates that if there are enough livers then everybody with tumour size below *S*_*M*_ would be transplanted. This should not be taken as an advantage of MC because (as can be seen from equation (14)) transplanting every patient in need is an exceptional case, that occurs when *S*_*M*_=*S*_*F*_. Remember that *S*_*M*_ is determined by law and *S*_*F*_ by chance, depending on the number of available grafts *F*, the incidence of HCC, *N* and the distribution of tumour size in these patients at presentation.

The MELD (Model for End stage Liver Disease) score has been selected as the most clinically appropriate tool for accurately predicting mortality in patients with chronic liver diseases [1,12-14]. However, the MELD score does not accurately predict survival in some patients, such as those with HCC. To enable patient with HCC to undergo LT at a rate similar to that for patients without HCC, additional points based on the number and size of the HCC nodules are assigned to patients with HCC on the waiting list; the intention is to match the risk of death for those with similar MELD scores but no HCC [15]. With this strategy, HCC patients have easier access to transplantation than non-HCC ones. In addition, this system does not allow for a dynamic assessment, which would be required to picture the current use of local tumor treatment.

Because of the paucity of donors organs, efforts have been made to optimize the effectiveness of LT through the application of strict criteria for selecting patients who have the greatest likelihood of prolonged survival after surgery. LT is a well-established treatment in a subset of patients with cirrhosis and HCC. The Milan criteria (single nodule up to 5 cm, up to three nodules none larger than 3 cm, with no evidence of extrahepatic spread or macrovascular invasion) have been traditionally accepted as standard of care. The introduction of MC improved 5 year survival post-LT for HCC from below 50% to greater than 70% [16,17]. However, some groups have proposed that these criteria are too restrictive, and exclude some patients from transplantation who might benefit from this procedure. Transplanting patients with tumors beyond the established criteria falls into two categories, those whose tumors are beyond the MC at presentation without the use of treatment prior to transplantation (expanded criteria), and those in whom treatment allows the MC to be fulfilled (down-staging). Currently, however, there is no international consensus regarding these approaches in clinical practice, as different populations such as Europeans, Americans or Asians have distinct HCC evolution and this should greatly influence the establishment of transplantation criteria [8,12].

Expanded Milan criteria (EMC) can be defined by the use of LT in recipients with tumors beyond the MC. The first description was published in 2001 by the group of the University of California, San Francisco (UCSF) [18]. In their study, 70 HCC-patients who underwent LT were retrospectively evaluated on the basis of explant analysis, not pre-transplant radiology. In the 60 cases with either a single nodule up to 6.5 cm, or up to three nodules none larger than 4.5 cm, and total tumor diameter no more than 8 cm the 5-year overall survival was 75.2%. Forty-six out of the 60 patients (76%) had tumors that were within the MC and these had a 5-year survival of 72%. Subsequently, a number of different EMC proposals have been described [19,20].

To optimize allocation of donated organs, Volk et al. [21] created a mathematical model focused on the lowest acceptable survival rate after LT for which the use of donor organs of standard quality could be justified and revealed that unless a 5-year survival of at least 61% could be achieved, performing LT for patients with tumors beyond MC put other patients without HCC at a risk of dying without LT [21]. This survival rate may increase to 71% in regions with severe organ shortage and reduced 25% in regions where shortage is not so acute. Samuel et al. [22] comment that this study has several limitations because it did not evaluate the use of donor organs of marginal quality, and it assumed that long-term survival after LT does not vary as a function of preoperative MELD score.

More recently, Tosa et al. [23] used a competitive risk model assessment, and, suggest a model for comparing the opportunities of receiving a graft for both HCC (deMELD) and non-HCC (MELD) patients on a common waiting list concluding that the allocation of deMELD (drop-out risk scores to HCC) has the potential to allow for a dynamic and combined comparison of opportunities to receive a graft for HCC and non-HCC patients on a common waiting list.

There is a lack of studies addressing these issues in the literature. In addition, the extrapolation of these findings to routine clinical practice is limited by our inability to accurately predict survival after LT for individual patients with HCC who do not meet the MC.

Finally, the methodology used in this paper explored the theoretical outcomes of HCC patients as a function of tumor size for transplantation, violating the limit proposed by the Milan Criterion. The model proposed was based on the calculation of mortality as a function of tumor size. Other indicators of clinical outcomes could be used instead of tumor size with the same model. In addition, other methods of analysis could be used to optimize the number of patients that could be transplanted, such as game theory [24] or non-binary logics like the theory of fuzzy sets [25-27]. This, however, will be subject of future work.

## Competing interests

The authors declare that they have no competing interests.

## Authors’ contributions

MA, FABC, LFL and EM conceived the study, designed the model based on existing research efforts, developed its computational implementation, carried out the model simulations and drafted the manuscript. EC, MNB and LACD participated in the design of the model providing guidelines from the clinical perspective, assisted in parameter estimation process, performed the evaluation of the model results, significantly contributed in the discussion section and suggested the future work extensions. EC, EM and LACD supervised the whole research, contributed to writing and improving the paper, suggested extensions and modifications and revised the manuscript critically. All authors read and approved the final manuscript.
